# Is there a perioperative circulatory side effect of intracameral epinephrine in hypertensive patients undergoing phacoemulsification?

**DOI:** 10.4103/0974-620X.71914

**Published:** 2010

**Authors:** Salima Bhallil, Idriss Benatiya Andalloussi, Oualid El Abdouni, Ibrahim Mahjoubi, Hicham Tahri

**Affiliations:** Department of Ophthalmology, University Hospital Hassan II, Fez, Morocco

Sir,

Cataract surgery requires adequate mydriasis which is achieved by topical administration or by intracameral injection of mydriatic agents. Since perioperative pupillary constriction may increase the risk of complications, mydriasis has to be maintained during surgery.

In practice, using topical mydriatics increase the waiting time for surgery. It is several folds longer than the surgical procedure time. Topically, administrated mydriatics increase the risk of cardiovascular side effects due to their significant systemic absorption. These effects are particularly perilous in high-risk groups such as patients with hypertension or cardiovascular diseases.[1,2]

Intracameral mydriatics are a rapid, effective, and safe alternative to topical mydriatics and can simplify preoperative routines. This regimen may reduce the risk for cardiovascular side effect.[2] However, there is no study concerning the circulatory effects of intracameral epinephrine in phacoemulsification cataract surgery in hypertensive patients.

We evaluated the systemic effects of intracameral epinephrine in medically treated arterial hypertensive patients. The epinephrine is introduced after corneal incision.

The statistical significance was estimated using χ^2^-test, and a *P* < 0.05 was considered significant.

The study group comprised of 300 cataracts patients, medically treated for hypertension, and scheduled for phacoemulsification. Two or three drops of 1 mL of epinephrine (1 mg/mL) diluted in 1 mL of normal saline was injected into the anterior chamber in all patients. One drop (50 μL) contained 25 μg of epinephrine. Topical mydriatics were not used. All procedures were performed by one surgeon. No epinephrine was added to the irrigating solution.

Peribulbar anesthesia using lidocaine 2% was achieved in all patients. Cataract surgery consists of a 3.2-mm superior clear corneal tunnel incision, intracameral injection of epinephrine, injection of viscoelastic material, capsulorhexis, hydrodissection, in the bag phacoemulsification, cortex aspiration, and insertion of foldable intraocular lens (IOL) in the capsular bag. The corneal incision was closed by hydration. A good mydriasis was maintained during surgery.

Preoperative, intraoperative, and postoperative pulse rate and blood pressure were recorded.

The mean of preoperative blood pressure was 117 ± 3/75 ± 2 mmHg. The pressure increased significantly after injection of local anesthesia. At the beginning of surgery, blood pressure values dropped down to preoperative values. Intraoperative and postoperative blood pressure after intracameral injection of epinephrine remained constant: 117 ± 2.5/65 ± 1.5 mmHg. No significant pulse rate changes were noted in the patients. There was no significant difference in the circulatory parameters before and after intracameral mydriatic injection. (*P* = 0.07).

Any cataract surgery practice requires sufficient mydriasis which is habitually achieved by topical mydriatic or by intracameral injection of mydriatics agents.[2]

Previous studies have concluded that intracameral mydriatic are an efficient substitute to traditional topical mydriatics with time saving and easier technique.[2,3] Systemic absorption of topical substances increases the risk for cardiovascular side effects.[4] On the other hand, the lower doses of mydriatics with intracameral technique may reduce the risk for cardiovascular side effect.[5]

To the best of our knowledge, this is the first study on the effect of intracameral injection of epinephrine on the blood pressure and pulse rate, in patients medically treated for arterial hypertension undergoing phacoemulsification.

In our study, the intraoperative blood pressure and pulse rate remained stable. We conclude that intracameral epinephrine is a safe alternative to topical mydriatics in phacoemulsification. The use of epinephrine in intracameral injection can simplify the preoperative routine.
